# (11*R*,12*S*)-16-Amino­tetra­cyclo­[6.6.2.0^2,7^.0^9,14^]hexa­deca-2(7),3,5,9(14),10,12-hexaen-15-ol

**DOI:** 10.1107/S1600536812026542

**Published:** 2012-06-20

**Authors:** Alaa A.-M. Abdel-Aziz, Adel S. El-Azab, Magda A. El-Sherbeny, Seik Weng Ng, Edward R. T. Tiekink

**Affiliations:** a Department of Pharmaceutical Chemistry, College of Pharmacy, King Saud University, Riyadh 11451, Saudi Arabia; b Department of Medicinal Chemistry, Faculty of Pharmacy, University of Mansoura, Mansoura 35516, Egypt; c Department of Organic Chemistry, Faculty of Pharmacy, Al-Azhar University, Cairo 11884, Egypt; d Department of Chemistry, University of Malaya, 50603 Kuala Lumpur, Malaysia; e Chemistry Department, Faculty of Science, King Abdulaziz University, PO Box 80203 Jeddah, Saudi Arabia

## Abstract

In the title compound, C_16_H_15_NO, the dihedral angle between the outer benzene rings is 51.88 (6)°, and each of the central six-membered rings has a boat conformation. The hy­droxy and amino groups are *syn*, and the hy­droxy H atom forms an intra­molecular O—H⋯N hydrogen bond. In the crystal, mol­ecules assemble *via* C—H⋯O and C—H⋯π inter­actions, consolidating a three-dimensional architecture.

## Related literature

For chiral ligands in asymmetric catalytic reactions, see: Yamakuchi *et al.* (2005 ▶). For the synthesis of the title compound, see: Hashimoto *et al.* (1998 ▶); Matsunaga *et al.* (2005 ▶). For a related structure, see: Abdel-Aziz *et al.* (2012 ▶).
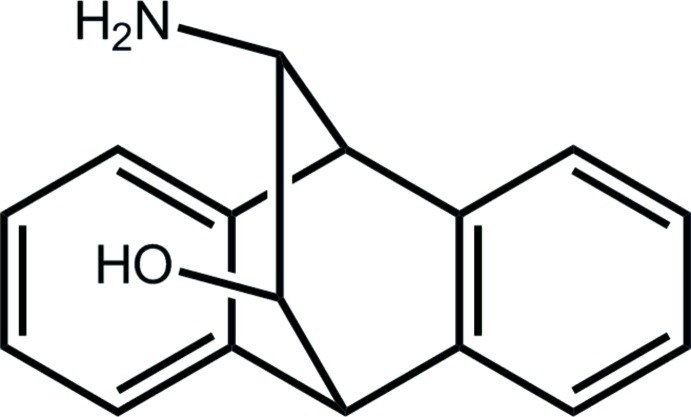


## Experimental

### 

#### Crystal data

C_16_H_15_NO
*M* *_r_* = 237.29
Monoclinic, 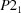

*a* = 8.6224 (2) Å
*b* = 7.1140 (1) Å
*c* = 10.0210 (2) Å
β = 106.707 (2)°
*V* = 588.74 (2) Å^3^
*Z* = 2
Cu *K*α radiation
μ = 0.65 mm^−1^
*T* = 100 K
0.40 × 0.30 × 0.20 mm

#### Data collection

Agilent SuperNova Dual diffractometer with an Atlas detector
Absorption correction: multi-scan (*CrysAlis PRO*; Agilent, 2012 ▶) *T*_min_ = 0.590, *T*_max_ = 1.000
4044 measured reflections
2375 independent reflections
2357 reflections with *I* > 2σ(*I*)
*R*_int_ = 0.011

#### Refinement

*R*[*F* ^2^ > 2σ(*F* ^2^)] = 0.029
*wR*(*F* ^2^) = 0.077
*S* = 1.06
2375 reflections
175 parameters
1 restraint
H atoms treated by a mixture of independent and constrained refinement
Δρ_max_ = 0.20 e Å^−3^
Δρ_min_ = −0.17 e Å^−3^
Absolute structure: Flack (1983 ▶), 1060 Friedel pairs
Flack parameter: 0.0 (2)

### 

Data collection: *CrysAlis PRO* (Agilent, 2012 ▶); cell refinement: *CrysAlis PRO*; data reduction: *CrysAlis PRO*; program(s) used to solve structure: *SHELXS97* (Sheldrick, 2008 ▶); program(s) used to refine structure: *SHELXL97* (Sheldrick, 2008 ▶); molecular graphics: *ORTEP-3* (Farrugia, 1997 ▶) and *DIAMOND* (Brandenburg, 2006 ▶); software used to prepare material for publication: *publCIF* (Westrip, 2010 ▶).

## Supplementary Material

Crystal structure: contains datablock(s) global, I. DOI: 10.1107/S1600536812026542/pv2558sup1.cif

Structure factors: contains datablock(s) I. DOI: 10.1107/S1600536812026542/pv2558Isup2.hkl

Additional supplementary materials:  crystallographic information; 3D view; checkCIF report

## Figures and Tables

**Table 1:** Hydrogen-bond geometry (Å, °) *Cg*1 and *Cg*2 are the centroids of the C1–C6 and C11–C16 benzene rings, respectively.

*D*—H⋯*A*	*D*—H	H⋯*A*	*D*⋯*A*	*D*—H⋯*A*
O1—H1*o*⋯N1	0.96 (3)	1.82 (2)	2.577 (2)	133 (2)
C5—H5⋯O1^i^	0.95	2.56	3.3506 (16)	141
C4—H4⋯*Cg*1^ii^	0.95	2.61	3.5064 (14)	158
C10—H10⋯*Cg*2^iii^	1.00	2.95	3.9212 (14)	164
C12—H12⋯*Cg*1^iii^	0.95	2.67	3.5159 (14)	149

Symmetry codes: (i) 
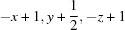
; (ii) 
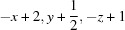
; (iii) 
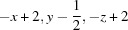
.

**Fig. 1.**
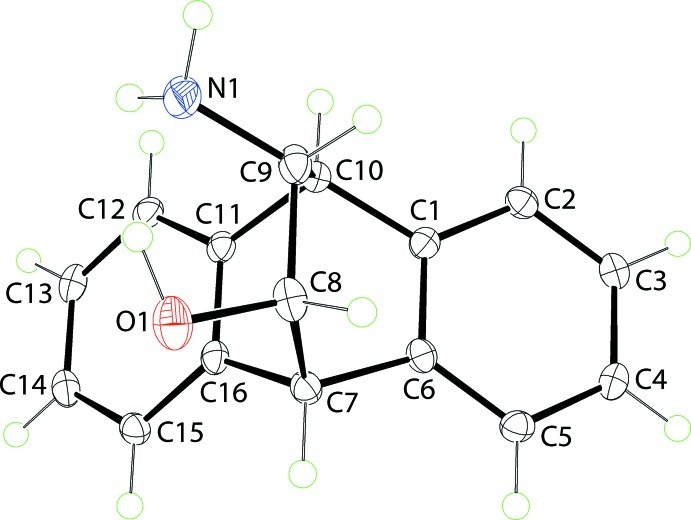
The molecular structure of the title compound showing the atom-labelling scheme and displacement ellipsoids at the 50% probability level.

**Fig. 2.**
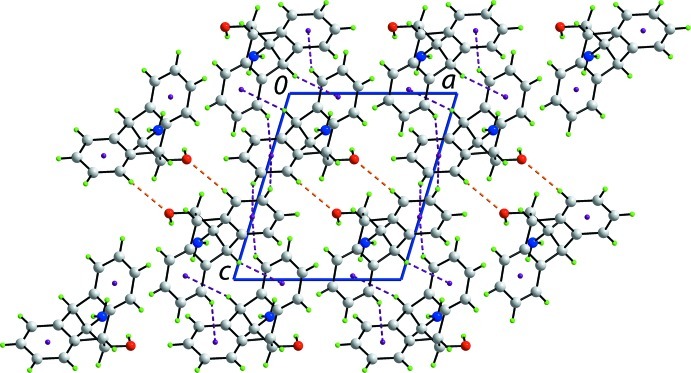
A view in projection down the b axis of the unit-cell contents for the title compound. The C—H···O and C—H···π interactions are shown as orange and purple dashed lines, respectively.

## supplementary crystallographic information

### Comment

The title compound was synthesized in relation to the development of chiral ligands for asymmetric catalytic reactions (Yamakuchi *et al.*, 2005) and in continuation of related structural studies (Abdel-Aziz *et al.*, 2012).

In the title molecule (Fig. 1), the dihedral angle between the (C1–C6) and (C11–C16) benzene rings is 51.88 (6)°. The dihedral angles between these planes and the central C7—C10 residue are 66.96 (5) and 61.17 (5)°, respectively. Each of the central six-membered rings (C1,C6–C10) and (C7–C10,C15,C15) has a boat conformation. The hydroxy and amino groups are *syn*, and the hydroxy-H atom is aligned to form an intramolecular O—H···N hydrogen bond (Table 1).

In the crystal packing, molecules assemble into a three-dimensional architecture *via* C—H···O and C—H···π interactions (Fig. 2 and Table 1).

### Experimental

The title compound was prepared following literature precedents (Hashimoto *et al.*, 1998; Matsunaga *et al.*, 2005). To 10,11,14,15-tetrahydro-9,10-[4,5]epoxazoloanthracen-13(9*H*)-one (2.0 ml), water (2 ml), ethanol (6 ml) and Ba(OH)_2_.8H_2_O (20 ml) were added. The mixture was heated at 413 K in a glass sealed tube for 72 h. The resulting solution was evaporated and extracted three times with chloroform (10 ml). The organic extract was dried and recrystallized from ethanol to afford the title compound.

### Refinement

Carbon-bound H-atoms were placed in calculated positions [C–H 0.95 to 1.00 Å, *U*_iso_(H) 1.2*U*_eq_(C)] and were included in the refinement in the riding model approximation. The hydroxy- and amino-H atoms were located in a difference Fourier map and were refined freely.

### Crystal data

**Table:**

C_16_H_15_NO	*F*(000) = 252
*M**_r_* = 237.29	*D*_x_ = 1.339 Mg m^−^^3^
Monoclinic, *P*2_1_	Cu *K*α radiation, λ = 1.54184 Å
Hall symbol: P 2yb	Cell parameters from 3312 reflections
*a* = 8.6224 (2) Å	θ = 4.6–76.4°
*b* = 7.1140 (1) Å	µ = 0.65 mm^−^^1^
*c* = 10.0210 (2) Å	*T* = 100 K
β = 106.707 (2)°	Prism, colourless
*V* = 588.74 (2) Å^3^	0.40 × 0.30 × 0.20 mm
*Z* = 2	

### Data collection

**Table:**

Agilent SuperNova Dual diffractometer with an Atlas detector	2375 independent reflections
Radiation source: SuperNova (Cu) X-ray Source	2357 reflections with *I* > 2σ(*I*)
Mirror monochromator	*R*_int_ = 0.011
Detector resolution: 10.4041 pixels mm^-1^	θ_max_ = 76.6°, θ_min_ = 4.6°
ω scan	*h* = −10→10
Absorption correction: multi-scan (*CrysAlis PRO*; Agilent, 2012)	*k* = −8→8
*T*_min_ = 0.590, *T*_max_ = 1.000	*l* = −12→7
4044 measured reflections	

### Refinement

**Table:**

Refinement on *F*^2^	Secondary atom site location: difference Fourier map
Least-squares matrix: full	Hydrogen site location: inferred from neighbouring sites
*R*[*F*^2^ > 2σ(*F*^2^)] = 0.029	H atoms treated by a mixture of independent and constrained refinement
*wR*(*F*^2^) = 0.077	*w* = 1/[σ^2^(*F*_o_^2^) + (0.0469*P*)^2^ + 0.1178*P*] where *P* = (*F*_o_^2^ + 2*F*_c_^2^)/3
*S* = 1.06	(Δ/σ)_max_ < 0.001
2375 reflections	Δρ_max_ = 0.20 e Å^−^^3^
175 parameters	Δρ_min_ = −0.17 e Å^−^^3^
1 restraint	Absolute structure: Flack (1983), 1060 Friedel pairs
Primary atom site location: structure-invariant direct methods	Flack parameter: 0.0 (2)

### Fractional atomic coordinates and isotropic or equivalent isotropic displacement parameters (Å^2^)

**Table:**

	*x*	*y*	*z*	*U*_iso_*/*U*_eq_	
O1	0.49638 (11)	0.50034 (15)	0.64491 (10)	0.0241 (2)	
N1	0.71887 (14)	0.29044 (18)	0.80145 (14)	0.0240 (3)	
C1	0.96576 (14)	0.71372 (17)	0.75647 (12)	0.0142 (2)	
C2	1.12329 (14)	0.73301 (18)	0.74782 (12)	0.0158 (2)	
H2	1.2081	0.6575	0.8038	0.019*	
C3	1.15501 (14)	0.86460 (19)	0.65592 (12)	0.0160 (2)	
H3	1.2620	0.8777	0.6488	0.019*	
C4	1.03187 (15)	0.97688 (19)	0.57454 (12)	0.0168 (2)	
H4	1.0557	1.0684	0.5142	0.020*	
C5	0.87298 (15)	0.95569 (18)	0.58111 (12)	0.0160 (2)	
H5	0.7882	1.0306	0.5244	0.019*	
C6	0.84091 (15)	0.82368 (17)	0.67170 (12)	0.0144 (3)	
C7	0.67531 (14)	0.77274 (18)	0.68480 (12)	0.0151 (2)	
H7	0.5883	0.8555	0.6263	0.018*	
C8	0.65031 (15)	0.56368 (19)	0.63948 (13)	0.0180 (3)	
H8	0.6555	0.5528	0.5414	0.022*	
C9	0.78995 (15)	0.43912 (18)	0.73668 (13)	0.0181 (3)	
H9	0.8514	0.3787	0.6773	0.022*	
C10	0.90784 (14)	0.57079 (17)	0.84339 (13)	0.0150 (3)	
H10	0.9998	0.4997	0.9070	0.018*	
C11	0.80732 (14)	0.67230 (17)	0.92286 (12)	0.0141 (2)	
C12	0.82683 (14)	0.66176 (18)	1.06492 (13)	0.0161 (2)	
H12	0.9093	0.5848	1.1225	0.019*	
C13	0.72441 (15)	0.76507 (18)	1.12256 (12)	0.0181 (3)	
H13	0.7377	0.7591	1.2199	0.022*	
C14	0.60311 (15)	0.87664 (18)	1.03815 (13)	0.0178 (3)	
H14	0.5346	0.9479	1.0782	0.021*	
C15	0.58155 (14)	0.88446 (17)	0.89508 (13)	0.0159 (2)	
H15	0.4978	0.9597	0.8374	0.019*	
C16	0.68295 (14)	0.78190 (18)	0.83726 (12)	0.0145 (2)	
H1o	0.526 (3)	0.384 (4)	0.696 (2)	0.048 (6)*	
H1n	0.720 (3)	0.316 (4)	0.891 (3)	0.060 (7)*	
H2n	0.772 (3)	0.179 (4)	0.803 (2)	0.043 (5)*	

### Atomic displacement parameters (Å^2^)

**Table:**

	*U*^11^	*U*^22^	*U*^33^	*U*^12^	*U*^13^	*U*^23^
O1	0.0167 (4)	0.0288 (6)	0.0270 (5)	−0.0084 (4)	0.0067 (4)	−0.0030 (4)
N1	0.0266 (6)	0.0152 (5)	0.0352 (6)	−0.0026 (5)	0.0170 (5)	−0.0005 (5)
C1	0.0170 (5)	0.0130 (6)	0.0131 (5)	−0.0007 (5)	0.0051 (4)	−0.0011 (4)
C2	0.0156 (5)	0.0163 (6)	0.0153 (5)	0.0009 (4)	0.0043 (4)	−0.0019 (4)
C3	0.0143 (5)	0.0184 (6)	0.0161 (5)	−0.0018 (5)	0.0059 (4)	−0.0029 (5)
C4	0.0200 (6)	0.0178 (6)	0.0141 (5)	−0.0025 (5)	0.0072 (4)	0.0004 (5)
C5	0.0178 (6)	0.0161 (6)	0.0135 (5)	0.0023 (5)	0.0036 (4)	0.0000 (4)
C6	0.0143 (5)	0.0156 (6)	0.0141 (5)	−0.0006 (4)	0.0051 (4)	−0.0023 (4)
C7	0.0127 (5)	0.0183 (6)	0.0146 (5)	0.0013 (5)	0.0042 (4)	0.0007 (5)
C8	0.0167 (6)	0.0211 (7)	0.0179 (6)	−0.0030 (5)	0.0075 (5)	−0.0039 (5)
C9	0.0201 (6)	0.0146 (6)	0.0229 (6)	−0.0029 (5)	0.0114 (5)	−0.0029 (5)
C10	0.0148 (5)	0.0141 (6)	0.0171 (6)	0.0006 (4)	0.0064 (4)	0.0017 (4)
C11	0.0149 (5)	0.0120 (6)	0.0162 (6)	−0.0023 (4)	0.0059 (4)	0.0001 (4)
C12	0.0168 (5)	0.0137 (6)	0.0177 (6)	−0.0019 (5)	0.0051 (4)	0.0020 (4)
C13	0.0222 (6)	0.0191 (6)	0.0152 (5)	−0.0057 (5)	0.0088 (5)	−0.0011 (5)
C14	0.0181 (6)	0.0158 (6)	0.0230 (6)	−0.0034 (5)	0.0113 (5)	−0.0040 (5)
C15	0.0132 (5)	0.0146 (6)	0.0206 (6)	−0.0005 (5)	0.0058 (4)	0.0002 (5)
C16	0.0142 (5)	0.0144 (6)	0.0164 (5)	−0.0027 (5)	0.0065 (4)	−0.0003 (5)

### Geometric parameters (Å, º)

**Table:**

O1—C8	1.4174 (15)	C7—C8	1.5518 (17)
O1—H1o	0.96 (3)	C7—H7	1.0000
N1—C9	1.4644 (17)	C8—C9	1.5841 (18)
N1—H1n	0.91 (3)	C8—H8	1.0000
N1—H2n	0.91 (3)	C9—C10	1.5586 (17)
C1—C2	1.3924 (16)	C9—H9	1.0000
C1—C6	1.4024 (16)	C10—C11	1.5177 (16)
C1—C10	1.5140 (16)	C10—H10	1.0000
C2—C3	1.3944 (18)	C11—C12	1.3867 (17)
C2—H2	0.9500	C11—C16	1.4017 (16)
C3—C4	1.3902 (17)	C12—C13	1.3954 (18)
C3—H3	0.9500	C12—H12	0.9500
C4—C5	1.3985 (17)	C13—C14	1.3895 (18)
C4—H4	0.9500	C13—H13	0.9500
C5—C6	1.3881 (17)	C14—C15	1.3928 (18)
C5—H5	0.9500	C14—H14	0.9500
C6—C7	1.5150 (16)	C15—C16	1.3869 (17)
C7—C16	1.5116 (15)	C15—H15	0.9500
			
C8—O1—H1o	100.4 (13)	C7—C8—H8	108.7
C9—N1—H1n	113.9 (17)	C9—C8—H8	108.7
C9—N1—H2n	110.8 (14)	N1—C9—C10	113.79 (11)
H1n—N1—H2n	107 (2)	N1—C9—C8	109.61 (10)
C2—C1—C6	120.00 (11)	C10—C9—C8	108.43 (10)
C2—C1—C10	126.23 (11)	N1—C9—H9	108.3
C6—C1—C10	113.60 (10)	C10—C9—H9	108.3
C3—C2—C1	119.17 (11)	C8—C9—H9	108.3
C3—C2—H2	120.4	C1—C10—C11	108.35 (10)
C1—C2—H2	120.4	C1—C10—C9	105.47 (10)
C2—C3—C4	120.76 (11)	C11—C10—C9	106.73 (10)
C2—C3—H3	119.6	C1—C10—H10	112.0
C4—C3—H3	119.6	C11—C10—H10	112.0
C3—C4—C5	120.25 (11)	C9—C10—H10	112.0
C3—C4—H4	119.9	C12—C11—C16	120.26 (11)
C5—C4—H4	119.9	C12—C11—C10	126.43 (11)
C6—C5—C4	119.06 (11)	C16—C11—C10	113.29 (10)
C6—C5—H5	120.5	C11—C12—C13	119.47 (11)
C4—C5—H5	120.5	C11—C12—H12	120.3
C5—C6—C1	120.73 (11)	C13—C12—H12	120.3
C5—C6—C7	126.05 (11)	C14—C13—C12	120.27 (11)
C1—C6—C7	113.11 (10)	C14—C13—H13	119.9
C16—C7—C6	108.00 (9)	C12—C13—H13	119.9
C16—C7—C8	107.41 (10)	C13—C14—C15	120.25 (11)
C6—C7—C8	105.01 (10)	C13—C14—H14	119.9
C16—C7—H7	112.0	C15—C14—H14	119.9
C6—C7—H7	112.0	C16—C15—C14	119.70 (11)
C8—C7—H7	112.0	C16—C15—H15	120.2
O1—C8—C7	110.26 (10)	C14—C15—H15	120.2
O1—C8—C9	110.68 (11)	C15—C16—C11	120.02 (11)
C7—C8—C9	109.82 (10)	C15—C16—C7	126.54 (11)
O1—C8—H8	108.7	C11—C16—C7	113.44 (10)
			
C6—C1—C2—C3	1.06 (17)	C2—C1—C10—C9	−113.84 (13)
C10—C1—C2—C3	176.01 (12)	C6—C1—C10—C9	61.39 (12)
C1—C2—C3—C4	0.59 (18)	N1—C9—C10—C1	−179.42 (10)
C2—C3—C4—C5	−1.72 (19)	C8—C9—C10—C1	−57.17 (11)
C3—C4—C5—C6	1.17 (18)	N1—C9—C10—C11	−64.31 (13)
C4—C5—C6—C1	0.48 (17)	C8—C9—C10—C11	57.94 (12)
C4—C5—C6—C7	−175.42 (11)	C1—C10—C11—C12	−128.68 (13)
C2—C1—C6—C5	−1.61 (17)	C9—C10—C11—C12	118.16 (13)
C10—C1—C6—C5	−177.17 (11)	C1—C10—C11—C16	52.62 (13)
C2—C1—C6—C7	174.79 (11)	C9—C10—C11—C16	−60.54 (13)
C10—C1—C6—C7	−0.77 (14)	C16—C11—C12—C13	−1.70 (18)
C5—C6—C7—C16	−129.68 (13)	C10—C11—C12—C13	179.68 (12)
C1—C6—C7—C16	54.15 (13)	C11—C12—C13—C14	0.43 (18)
C5—C6—C7—C8	115.94 (13)	C12—C13—C14—C15	0.78 (18)
C1—C6—C7—C8	−60.23 (12)	C13—C14—C15—C16	−0.72 (18)
C16—C7—C8—O1	66.32 (12)	C14—C15—C16—C11	−0.55 (17)
C6—C7—C8—O1	−178.88 (10)	C14—C15—C16—C7	179.28 (12)
C16—C7—C8—C9	−55.90 (12)	C12—C11—C16—C15	1.77 (17)
C6—C7—C8—C9	58.89 (12)	C10—C11—C16—C15	−179.44 (11)
O1—C8—C9—N1	1.54 (14)	C12—C11—C16—C7	−178.08 (11)
C7—C8—C9—N1	123.51 (11)	C10—C11—C16—C7	0.71 (14)
O1—C8—C9—C10	−123.22 (11)	C6—C7—C16—C15	126.02 (13)
C7—C8—C9—C10	−1.25 (13)	C8—C7—C16—C15	−121.19 (13)
C2—C1—C10—C11	132.18 (12)	C6—C7—C16—C11	−54.14 (14)
C6—C1—C10—C11	−52.59 (13)	C8—C7—C16—C11	58.65 (12)

### Hydrogen-bond geometry (Å, º)

Cg1 and Cg2 are the centroids of the C1–C6 and C11–C16 benzene rings, respectively.

**Table:**

*D*—H···*A*	*D*—H	H···*A*	*D*···*A*	*D*—H···*A*
O1—H1*o*···N1	0.96 (3)	1.82 (2)	2.577 (2)	133 (2)
C5—H5···O1^i^	0.95	2.56	3.3506 (16)	141
C4—H4···*Cg*1^ii^	0.95	2.61	3.5064 (14)	158
C10—H10···*Cg*2^iii^	1.00	2.95	3.9212 (14)	164
C12—H12···*Cg*1^iii^	0.95	2.67	3.5159 (14)	149

Symmetry codes: (i) −*x*+1, *y*+1/2, −*z*+1; (ii) −*x*+2, *y*+1/2, −*z*+1; (iii) −*x*+2, *y*−1/2, −*z*+2.
